# Screening Antioxidant Components in Yiwei Decoction Using Spectrum-Effect Relationship and Network Pharmacology

**DOI:** 10.1155/2024/5514265

**Published:** 2024-10-16

**Authors:** Lei Zhang, Wei Zhu

**Affiliations:** Kunshan Hospital of Traditional Chinese Medicine, Kunshan, China

**Keywords:** antioxidant activity, bioinformatics, fingerprint, molecular docking, multivariate statistical analysis, Yiwei Decoction

## Abstract

Yiwei decoction (YWD) is a classic prescription with the function of nourishing stomach yin. In this study, the effective components of antioxidant activity of YWD and its possible mechanism were discussed from the point of view of spectral effect relationship and network pharmacology. Firstly, the fingerprints of 10 batches of YWD were established by UPLC-PDA technique, and the 1,1-diphenyl-2-picryl-hydrazyl radical (DPPH) scavenging rate and total antioxidant capacity (T-AOC) were used as the indicators for antioxidant activity in vitro. Then, the spectral effect relationship between the fingerprint profiles and antioxidant capacity was analyzed through grey relational analysis (GRA) and orthogonal projections to latent structures (OPLS). In addition, network pharmacology was employed to predict the potential mechanisms of YWD in the treatment of antioxidant-related diseases. The spectrum-effect relationship indicated that three common peaks were likely to be the most decisive active components, identified as verbascoside, psoralen, and vitexin, respectively. Based on network pharmacology analysis, a total of 83 target genes shared by the active components and antioxidant-related diseases were collected. AKT1, HSP90AA1, SRC, CASP3, and MTOR were closely related to antioxidant therapy and considered as core therapeutic targets. The potential mechanisms of YWD were obtained through gene ontology (GO) and Kyoto Encyclopedia of Genes and Genomes (KEGG). Finally, molecular docking simulations were conducted to evaluate the binding activities between the core therapeutic targets and corresponding compounds. The excellent core protein-compound complexes obtained by molecular docking were simulated by molecular dynamics simulation. The results showed that the active compounds had good binding ability with the selected targets. This study successfully identified the effective components of YWD and predicted the potential targets and pathways, which provided a new idea for the application of YWD in the treatment of antioxidant stress in the future. In addition, the potential active components provide valuable implications for drug screening of related diseases.

## 1. Introduction

The imbalance between excessive production of reactive oxygen species (ROS) and limited antioxidant defense capacity within the organism leads to various detrimental processes, which is known as oxidative stress [1]. In this complex equilibrium system, ROS, as the main medium of oxidative stress, can be neutral molecules (such as hydrogen peroxide), ions (such as superoxide anions), or free radicals (such as hydroxyl radicals), and play a role by regulating cell signal cascades [2, 3]. If this imbalance can be corrected, excessive ROS will lead to the modification of cellular macromolecules, thereby destroying cellular proteins, lipids, and DNA, resulting in irreversible cell damage. This regulation of oxidative stress at the cellular level is involved in DNA repair, cell proliferation, and gene expression of downstream targets of antioxidants [4, 5], directly affecting tissues, organs and ultimately the function of the entire body [6], and involving the occurrence of various pathological conditions, such as cardiovascular disease [7], neurodegenerative disease [8], osteoporosis [9], diabetes and Alzheimer's disease [10], cancer [11], and so on. In the past few decades, significant efforts have been made to explore the potential benefits of antioxidants [12], and gradually focus on plants that are both medicine and food. They have the functions of antioxidation, antitumor, immune regulation, improving memory, protecting the cardiovascular system, and improving diabetes [13].

As a key part of complementary and alternative medicine, the theory of homology of medicine and food has a long history in China. This theory holds that a large number of Chinese medicinal herbs can be used both medicinally and edible, such as chrysanthemum, *Lonicera Japonica*, Flos, containing flavonoids, organic acids, terpenes, and other active ingredients, with various medicinal values such as anti-inflammatory, antitumor, antioxidation, and so on [14]. In modern times, some traditional Chinese medicine or its components have been widely used in functional foods or health products [15], characterized by safety, efficacy, and low side effects [16]. YWD is a classic prescription composed of four traditional Chinese medicines and rock sugar for auxiliary function that are homologous to food and medicine, which has antitumor effect mediated by spleen exocrine [17]. Previous studies have revealed that catalpol of *Rehmannia glutinosa* [18], Ophiopogonin D of Ophiopogonis Radix [19], and the aqueous extract of *Polygonatum odoratum* [20] all showed inhibitory effects on oxidative stress. As the widespread application of water extraction technology in the production of traditional Chinese medicine, these water-soluble compounds in YWD can be easily obtained through traditional methods [21, 22]. However, due to the diversity of the origin of traditional Chinese medicine and the complexity of chemical composition, it is difficult to control its quality by single detection mothed. Fortunately, the existence of fingerprint profiles provides insights into the study of mixtures. Despite the advantages and disadvantages of different methods, the combined use of ultra-high-performance liquid chromatography (UPLC) and photodiode array (PDA) has been proved to have features such as good separation efficiency, high accuracy, and short analysis time [23]. In addition, due to the colored and stable characteristics of DPPH-free radicals, DPPH scavenging test has become an easy and accurate method to measure the total antioxidant capacity (T-AOC) of plant or herbal extracts and to screen antioxidants [24]. Sampling enzyme meters to detect T-AOC is simple, rapid, and sensitive. It is becoming a technical tool to quantify the antioxidant capacity of bioactive substances [25]. More importantly, a modern multivariate statistical analysis method, including orthogonal partial least squares regression (OPLS) [26] and grey relational analysis (GRA) [27], has been widely applied for analyzing the correlation between regression coefficients, Variable Importance in Projection (VIP) contributions, and their correlation with spectral effects. Active compounds closely related to special effects can be effectively screened for further pharmacological activity evaluation in vitro or in vivo. The unknown action mechanism of the screened active compounds can be solved by rapid and comprehensive network pharmacology and molecular docking methods [28].

In this study, the fingerprint profiles and antioxidant activity of 10 batches of YWD were evaluated by UPLC and antioxidant determination, and the spectral effect relationship of YWD was established by a series of correlation analysis. Finally, the possible mechanism of YWD in the treatment of oxidative stress-related diseases was revealed by network pharmacological analysis and molecular docking technology.

## 2. Materials and Methods

### 2.1. Instrument

The Waters ACQUITY UPLC system was used for fingerprint analysis, equipped with a vacuum degasser, quaternary pump, sample manager, and PDA detector (Waters, Milford, Massachusetts, USA). MassLynx V4.1 software was used for instrument control and data acquisition. The iMark enzyme labeling instrument was obtained from BIO-RAD (USA). The BT25S (0.01 mg) and SOP (0.1 mg) electronic balances were obtained from Sedorius Scientific Instruments (Beijing, China). KQ5200DE CNC ultrasonic cleaner was purchased from Kunshan Ultrasonic Instrument Co., Ltd. The TGL-16G high-speed desktop centrifuge was obtained from Shanghai Anting Scientific Instrument Factory. The Mill-Q ultra-pure water dispenser was purchased from Millipore (USA).

### 2.2. Materials and Reagents

YWD is composed of *Rehmannia glutinosa*, Ophiopogonis Radix, *Plygonatum odoratum*, *Glehnia littoralis*, and rock sugar, which is combined in a ratio of 5:5:1.5:3:1. Ten batches of YWD pieces of traditional Chinese medicine decoction (S1–S10) were purchased from various regions in China, as shown in Table 1. They were identified and confirmed by professional senior Chinese medicine experts. Rock sugar for auxiliary function was purchased from Suzhou Tianling Chinese Medicine Co., Ltd. verbascoside (number: MUST-23030717), psoralen (number: MUST-23020719), and vitexin (number: MUST-22092707) were purchased from Chengdu Mansite Biotechnology Co., Ltd. All reference standards were of chromatographic purity with a quality fraction of ≥ 98.0%. Acetonitrile, methanol, and formic acid were of chromatographic purity, while other reagents were of analytical purity. DPPH (number: D807297) was purchased from Shanghai Maclin Biochemical Technology Co., Ltd., and T-AOC (number: BC1315) was purchased from Beijing Solabo Biotechnology Co., Ltd.

### 2.3. Establishment of Fingerprint Profiles

#### 2.3.1. Chromatographic Conditions

An Agilent Poroshell 120EC C18 column (2.1 mm × 100 mm, 1.9 *μ*m) was used with acetonitrile (A)-0.1% formic acid-aqueous solution (B) as the mobile phase for gradient elution. The gradient elution procedure was as follows: 0∼10 min, 5% ⟶ 15%A. 10∼17 min, 15% ⟶ 18%A; 17∼33 min, 18% ⟶ 33%A; 33∼38 min, 25% ⟶v40%A; 38∼43 min, 40% ⟶ 80%A; 43–50 min, 80% ⟶ 5%A. The column temperature was 30°C, the sample disk temperature of the automatic injector was 30°C, the detection volume flow rate was 0.1 mL·min^−1^, the injection volume was 1 *μ*L, and the detection wavelength was 330 nm.

#### 2.3.2. YWD Preparation

The traditional Chinese medicines were weighed according to the prescription proportion, soaked in water at 1:20 (w/v) for 30 min, heated to boiling and kept slightly boiling for 60 min. Then filtered while hot and the dregs were boiled with water at 1:10 (w/v) again, keeping slightly boiling for 30 min, filtered and combined with primary filtrate, concentrated under reduced pressure to 200 mL. Precisely transferred 8 mL of the concentrated solution to the 25 mL measuring bottle, added methanol to the mark, precisely calibrated the mass, sonicated (500 W, 40 kHz) for 10 min, centrifuged (10,000 rpm) for 10 min, and marked up the mass after cooling. The supernatant obtained through 0.22 *μ*m filter membrane is taken as the test solution.

#### 2.3.3. Preparation of Reference Solution

Precise amounts of verbascoside, psoralen, and vitexin of 6.20, 3.18, and 4.98 mg, respectively, were separately weighed and placed into 5 mL volumetric flasks. Methanol was added to dissolve the compounds and the solutions were diluted to the mark, followed by ultrasonication and thorough mixing to obtain individual reference solutions. Subsequently, 0.5 mL, 0.5 mL, and 1 mL of each reference solution were taken and transferred into 50 mL volumetric flask. Methanol was added to the volumetric flask, shake well, and then get the mixed reference solution.

#### 2.3.4. Methodological Investigation of Fingerprint Analysis

The methodology validation involved examining the retention time and peak area of the samples separately. Precision was studied by calculating the relative standard deviation (RSD) of six consecutive injections of the same sample. Repeatability was investigated by determining the RSD value of six replicate injections of parallelly prepared samples. Stability was assessed by measuring the RSD value of the same sample injected at different time points (0, 2, 4, 8, 12, and 24 h).

#### 2.3.5. Establishment of Common Pattern of Fingerprint and Evaluation of Similarity

Ten batches of extracted solution of YWD were taken as test samples and determined in accordance with the conditions under “2.3.1.” The chromatograms were recorded and imported into the “Similarity evaluation system for chromatographic fingerprint of traditional Chinese Medicine (Version 2012.130723).” The time window was set as 0.1 min, S6 as the reference fingerprint, multipoint correction, peak matching, and median method were employed sequentially to generate the control fingerprint and calculate the similarity. A volume of the mixed control solution under “2.3.3” was precisely absorbed, and the chromatographic conditions under “2.3.1” were used to determine the retention time of the control samples and identify characteristic peaks for comparison.

### 2.4. Antioxidation Assay

#### 2.4.1. Preparation of Test Sample Solution

After filtering the concentrated solution of “2.3.2,” absorb 0.1 mL and transfer it into a 10 mL capacity bottle, dilute the volume to the mark with water, store at 4°C, and set aside for later use.

#### 2.4.2. Determination of DPPH Radical Scavenging Activity

Precisely weigh 2.50 mg of DPPH in 25 mL volumetric flask, fill with methanol up to the mark, shake well, and store in the dark to obtain the free radical solution of 0.10 mg/mL. The solutions of 1, 2, 3, 4, and 5 mL were precisely absorbed, and methanol was diluted to 10 mL to obtain a series of DPPH solution concentrations of 0.01, 0.02, 0.03, 0.04, and 0.05 mg/mL. Measured the absorbance values at 517 nm using enzyme labeling instrument, and performed triplicate measurements in parallel for each concentration.

The standard curve of DPPH was drawn with concentration (mg/mL) as abscissa and absorbance (A) as ordinate. Then, all the solutions were precisely absorbed and placed on the 96-well enzyme plate, and the absorbance was measured after a light-avoided reaction for 30 min, and the DPPH· clearance rate was calculated. Then, all the solutions were precisely absorbed and placed on 96-well enzyme label plate, and the absorbance was measured after 30 min of light avoidance reaction, and the DPPH· clearance rate was calculated. DPPH·scavenging rate (%) = [1 − (Asample − Acontrol)/Astandard] × 100% [11], where Asample is the absorbance of 100 *μ*L sample solution added with 100 *μ*L DPPH solution, Acontrol is the absorbance of 100 *μ*L sample solution reacted with 100 *μ*L methanol, and Astandard is the absorbance of 100 *μ*L methanol added to 100 *μ*L DPPH solution.

#### 2.4.3. Determination of T-AOC

Prepared the reaction mixture according to the instructions of the T-AOC kit, accurately absorbed 180 *μ*L of the mixture and 18 *μ*L of distilled water into the determination tube, then added 6 *μ*L of YWD test sample solution, mixed and reacted 10 min at room temperature, then absorbed 200 *μ*L into the 96-well enzyme plate, replaced the sample solution with 6 *μ*L of distilled water in the blank group, and determined the absorbance at 593 nm. Each concentration was measured in parallel for 3 times, and the T-AOC (*μ* mol/mL) was calculated.

### 2.5. Spectrum-Effect Relationship Analysis

#### 2.5.1. GRA Analysis

Data of DPPH·scavenging rate and T-AOC of YWD solution with a mass concentration of 2.89 mg/mL (measured by drug dose) and 10 batches of common peak data with good separation were imported into SPSSAU for correlation analysis. Due to the different dimensions of the data, normalization was required to eliminate the bias caused by dimensionality. Therefore, the mean normalization method was chosen to process the imported data, with a resolution coefficient of 0.5. The efficacy indicators of antioxidant activity (free radical scavenging rate, T-AOC) in different batches were taken as parent sequences, and the common peak areas of different batches were selected as subsequences. The software automatically processed and calculated the correlation coefficient.

#### 2.5.2. OPLS Analysis

In order to further clarify the correlation between chemical components and efficacy of YWD, the normalized area of 15 major chromatographic peaks of YWD solution with mass concentration of 2.89 mg/mL as independent variable (*X*) and the pharmacodynamic data of scavenging rate of DPPH· and T-AOC as dependent variable (*Y*) were introduced into SIMCA14.1 software for OPLS analysis and related models were established. The contribution of *X* to *Y* was judged based on the VIP index, which indicates the importance of variables. Generally, variables with larger VIP values are considered to have greater contributions.

### 2.6. Network Pharmacology Prediction

#### 2.6.1. Target Prediction and Screening

Based on the judgment of spectral effect relationship and the detection of standard samples, we obtained the structural information of the identified peak components one by one through the PubChem database (https://pubchem.ncbi.nlm.nih.gov/). And then imported them into the SwissTargetPrediction platform (https://www.swisstargetprediction.ch/) for prediction. Only targets with a confidence level ≥ 0.1 were selected for further analysis. Using “oxidative stress” as the disease keyword from GeneCards database (https://www.genecards.org/), and after the Uniprot data (https://www.uniprot.org/) normalization, the disease targets and the targets of the common peak component were matched. Next Venn diagram was constructed using online bioinformatics analysis and visual cloud platform (bioinformatics.com.cn), and the potential action targets of YWD were obtained.

#### 2.6.2. PPI Analysis

Submitted all gene symbols of the obtained potential protein targets to the STRING database (https://string-db.org/), and then imported into the network visualization software Cytoscape 3.7.2 to obtain the PPI network. Further, use the cytoHubba analysis function to obtain the Hub targets. The larger the degree value of the target, the larger its shape, indicating a tighter connection with other targets in the network.

#### 2.6.3. GO and KEGG Analysis

GO and KEGG analysis were performed on potential effective targets of WYD using Metascape platform, and the top 20 biological processes and signaling pathways with *p* < 0.001 were selected for further analysis and visualized using online bioinformatics analysis and visualization cloud platform to generate bubble plots.

#### 2.6.4. “Component-Target-Pathway” Network

The “component-target-pathway” network was constructed by Cytoscape3.7.2 software, so as to better reveal the regulation mechanism of the three main components on action targets and related pathways.

#### 2.6.5. Molecular Docking

In order to explore the interaction and affinity between active compounds screened through spectrum-effect relationship and key targets, we adopted the strategy of molecular docking to confirm the reliability of therapeutic targets. In this study, the structure of the key active ingredients of the drug was downloaded from the PubChem database and saved them as SDF files. The small molecular mechanics was optimized by 3D software and saved as MOL files. The PDBID of disease-related genes was queried and the three-dimensional structures of the target protein IDs were downloaded from PDB database. The receptor protein was dehydrated, hydrogenated, measured using AutoDockTools1.5.7 software and stored in pdbqt format, and the small molecular ligands were also saved in pdbqt format [29]. Next, molecular docking experiments were carried out with AutodockVina1.1.2. In general, binding energy is used to assess the binding efficiency between therapeutic protein targets and candidate molecules. The lower the binding energy is, the more stable the structure is. The candidate targets were screened with the binding energy < −5 kcal/mol as the threshold [30], and the visual analysis was carried out by Pymol and Discovery Studio software.

#### 2.6.6. Molecular Dynamics Simulation

In order to analyze the binding affinity between protein targets and active compounds obtained by molecular docking, MD simulation was performed using the Gromacs2019.6 program under constant temperature and constant pressure and periodic boundary conditions. The amber99sb-ildn protein force field and TIP3P water model were applied. During the MD simulation, all involved constraints were applied using the LINCS algorithm, with an integration time step of 2 fs. The electrostatic interactions were calculated using the particle-mesh Ewald (PME) method. The nonbonded interactions were truncated at 10 Å, with updates every 10 steps [31]. The simulation temperature was controlled at 300 K using the Berendsen temperature coupling method, and the pressure was controlled at 1 bar using the Parrinello–Rahman method. Firstly, energy minimization was performed on both systems using the steepest descent method to eliminate atomic contacts that were too close. Then, the systems were heated to 300 K within 100 ps. Finally, 100 ns MD simulations were performed on the systems, with conformations saved every 20 ps. The simulation results were visualized using the built-in programs in Gromacs and Pymol. The calculation and decomposition of free energy were performed using MM-PBSA [32].

## 3. Results and Discussion

### 3.1. Fingerprint Pattern Results

#### 3.1.1. Optimization of Chromatographic Conditions

In order to optimize the UPLC method, we investigated the effects of different column temperatures (25°C, 30°C), different mobile phase systems (acetonitrile-water, acetonitrile-0.1% formic acid water), and different wavelengths (254, 296 and 330 nm). The results showed that both peak width and resolution were excellent at 30°C, and the peak shape in 0.1% formic acid water was better than that of distilled water, as formic acid promoted the ionization of compounds [33]. Moreover, Supporting Figure S1 clearly shows the overall detection at wavelengths of 254, 296 and 330 nm, we found that more components were detected at 330 nm wavelength and showed strong absorption, so this ultimately became the chromatographic condition for this analysis.

#### 3.1.2. Methodological Verification of Fingerprint

Through calculating the relative retention time and relative peak area of each common peak, the precision results indicated that the RSD of the relative retention time of each common peak was less than 1.50% and the RSD of the relative peak area was less than 3.00%, indicating good instrument precision. The repeatability test results showed that the RSD of the relative retention time of each common peak was less than 0.26% and the RSD of the relative peak area was less than 4.75%, indicating good repeatability of the method and its suitability for the fingerprint detection of YWD. The stability investigation results demonstrated that the RSD of the relative retention time for each common peak was less than 0.44% and the RSD of the relative peak area was less than 4.92%, indicating the stability of the test sample solution within 24 h. It could be seen that the established fingerprint was effective.

#### 3.1.3. Establishment of Fingerprints and Identification of Common Peaks

**Figure 1:**
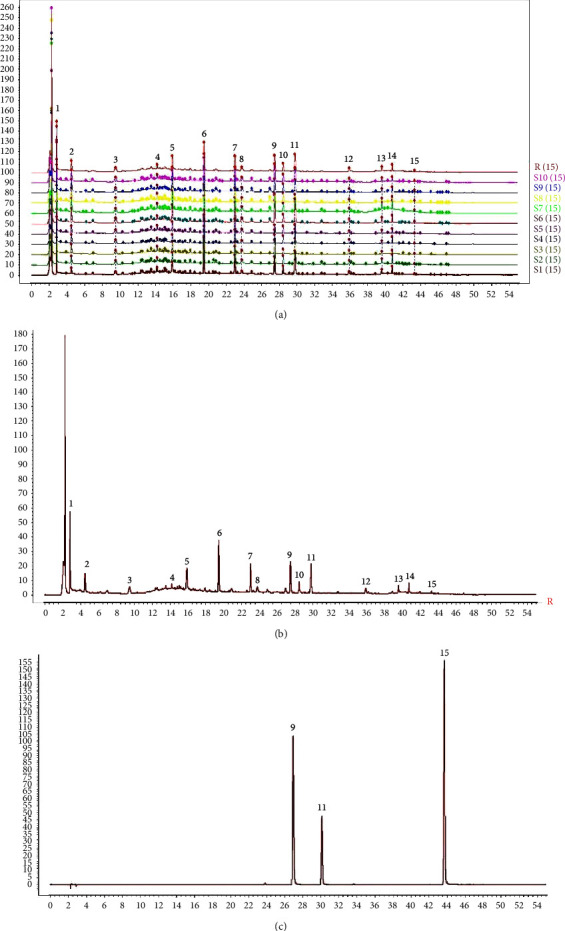
UPLC fingerprints of YWD and their reference (the detection wavelength was set at 330 nm, and the time is expressed in minutes). (a) 10 batches of YWD; (b) reference fingerprints; (c) standard fingerprint.

After detection using the selected chromatographic conditions and similarity analysis, the superimposed fingerprint chromatograms of 10 batches of YWD reference samples were obtained (Figure 1(a)). Based on the matching information, 15 chromatographic peaks were identified as common peaks, which could represent the samples well (Figure 1(b)). Through the identification of the chromatogram, it was determined that the 9th peak was vitexin, the 11th peak was verbascoside, and the 15th peak was psoralen (Figure 1(c)).

#### 3.1.4. Similarity Evaluation

Among them, the resolution and peak shape of Peak 9 were good, so it was taken as the reference peak to calculate the RSD value of the relative retention time of the common peak of 10 batches of YWD samples, that were all less than 1%. However, the RSD values of the relative peak areas were relatively large, indicating certain differences in the content of the main components among YWD samples from different regions (Tables 2 and 3). The results of similarity evaluation showed that except for S2 which had a similarity lower than 0.9, the similarity of other batches was similar (all greater than 0.94), indicating that the processing methods and storage conditions of various traditional Chinese medicine were stable, but the batches from different provinces were slightly different. It was speculated that the differences in the contents of different components of the same medicinal materials were due to different natural conditions in different regions, while the varieties of medicinal materials in the same region were relatively similar, such as S3 and S4, S9 and S10 (Table 4). However, although the fingerprint spectrum could reflect the content of various index components in medicinal materials, it could not make an inherent quality correlation between the components and pharmacological activity, therefore, it is necessary to combine multiple factor statistical methods for evaluation [34].

### 3.2. Antioxidant Activity Results

The single pair electrons of DPPH, due to their highly stable properties, are commonly used as a test substrate to evaluate the antioxidant activity of free radical scavengers. As shown in Table 5, S7 exhibited the strongest DPPH radical scavenging activity, while S3 and S8 with weaker antioxidant activity. There were certain differences in antioxidant activity among different batches. T-AOC test found that various antioxidants in 10 batches of YWD extract exhibited overall T-AOC. Among them, S10 showed the strongest activity, while S5 and S6 exhibited weaker activity, which may be closely related to the intensity and action tendency of related components. Although previous studies have confirmed the antioxidant activity of individual herbs of YWD prescription, the spectrum-effect relationship between the overall composition and antioxidant activity of YWD has not been elucidated. The results of antioxidant activity indicated that for 10 batches of YWD extracts, the antioxidant trends shown by the two methods were not consistent, suggesting that currently no method could comprehensively evaluate the T-AOC of the samples, while this study combined with two different antioxidant tests could better evaluate the overall antioxidant activity of the samples. Furthermore, we will integrate the chemical fingerprint and pharmacological activity levels to comprehensively evaluate the superiority and inferiority of YWD's T-AOC.

### 3.3. Results of Spectrum-Effect Relationship

In GRA, we used the scavenging rate of DPPH-free radicals and the measured value of T-AOC as indicators to investigate the antioxidant activity of different batches of YWD in order to screen out the antioxidant components. As can be seen from Table 6, the correlation degree between the labeled common peaks and DPPH-free radical scavenging rate and T-AOC is greater than 0.5, indicating that all peaks were involved in the antioxidant process and finally form a positive or negative effect. Among them, the common peaks *X*_6_,  *X*_8_,  *X*_13_,  *X*_14_, and X_15_ had a similar effect on the clearance rate of DPPH radicals and T-AOC. However, there were significant difference in the effects of *X*_3_,  *X*_5_,  *X*_10_,  *X*_11_, and *X*_12_, indicating that the types of free radicals cleared by various components are different. It should be noted that although GRA provided a practical and efficient method for us to identify bioactive substances from complex TCM systems [35], it was still unable to determine the positive or negative effects of the antioxidant level of common peaks. Therefore, this experiment combined with OPLS to jointly screen active components to improve the accuracy of the results. Taking the peak area of 10 batches of common peaks of YWD as independent variables, the scavenging rate of DPPH-free radicals, and T-AOC level of YWD solution as dependent variables, OPLS analysis was carried out by SIMCA14.1 software, and the models were established, respectively. The fitting regression equation of DPPH-free radical scavenging rate was as follows: *Y* = −0.0061*X*_1_+0.0962*X*_2_ − 0.0128*X*_3_ − 0.0073*X*_4_ + 0.0060*X*_5_+0.1202*X*_6_+0.0867*X*_7_+0.1376*X*_8_ + 0.0819*X*_9_+0.0732*X*_10_+0.0732*X*_11_+0.0137*X*_12_+0.1056*X*_13_+0.1041*X*_14_+0.0759*X*_15_, and T-AOC level as follows: *Y* = −0.6470*X*_1_ − 0.4301*X*_2_+0.1614*X*_3_+0.3232*X*_4_ + 0.0574*X*_5_ − 0.2917*X*_6_ − 0.0770*X*_7_+0.1982*X*_8_+0.1847*X*_9_+0.1697*X*_10_ + 0.1697*X*_11_ − 0.0746*X*_12_+0.2267*X*_13_+0.0942*X*_14_+0.2386*X*_15_.

**Figure 2:**
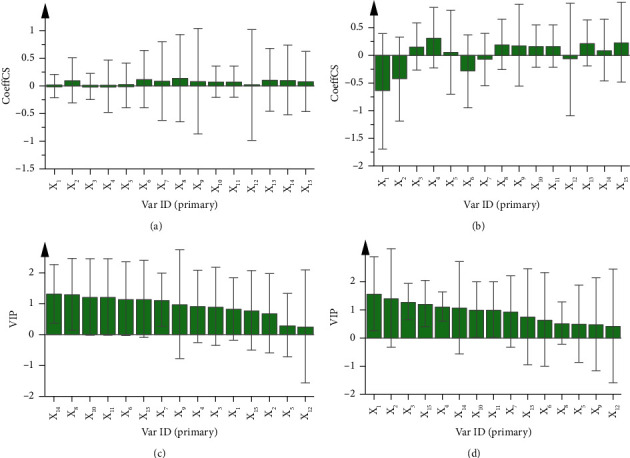
The analysis of pharmacological experiments by OPLS. OPLS regression coefficients (a, b) and VIP value (c, d) of the 15 compounds analyzed. (a, c) belonged to DPPH, (b, d) belonged to T-AOC.

In the two equations, the regression coefficient represented the contribution of the respective variables to the free radical scavenging rate and T-AOC. The larger the coefficient, the greater the contribution to the efficacy. A positive value indicates a positive correlation with the effect, while a negative value represented a negative correlation (Figures 2(a) and 2(b)). Since the standard regression coefficient only reflected the promoting or inhibiting effect between components and indicators, and could not measure the importance of different variables, it was necessary to further calculate the VIP values of different chemical components to the overall antioxidant level. The greater the value, the greater the contribution. When the VIP value is greater than 1, it is generally considered as an important influence index [36]. It could be observed from Figures 2(c) and 2(d) that for DPPH radical, the components with VIP greater than 1 and positive regression coefficient included peak 6, peak 7, peak 8, peak 10, peak 11, peak 13, and peak 14, while peak 9 was also very close to 1. For T-AOC, the components with VIP greater than 1 and positive regression coefficient included peak 3, peak 4, peak 10, peak 11, peak 14, and peak 15, while peak 7 is also very close to 1. Therefore, the main components of the comprehensive screening were peak 10, peak 11, and peak 14. Considering the results of GRA and the positive scavenging effect of peak 9 on DPPH radicals, as well as the significant contribution of peak 15 to T-AOC, we believed it was meaningful to further explore the action mechanism of identified peaks 9, peak 11, and peak 15.

Here, phenylethanol glycoside is a widely distributed class of natural water-soluble compounds in the plant kingdom, which possesses significant biological characteristics. Verbascoside, as a representative phenylethanol glycoside, exhibited beneficial pharmacological activities for human health, including antioxidant, anti-inflammatory, and anti-tumor properties, as well as many wound healing and neuroprotective properties [37]. A large animal model study found that verbascoside improved embryonic development by protecting oocytes against oxidative stress [38]. Furthermore, the antioxidant effect of verbascoside on Fe(2+)/ADP-induced rat liver mitochondrial liposomes (RLML) lipid peroxidation suggested that the inhibitory activity of phenylethanol glycoside-like compounds primarily originates from the chain-breaking mechanism of free radicals [39]. Vitexin is a kind of apigenin flavonoid glycoside, which acts as an antioxidant against ROS, lipid peroxidation, and other oxidative damage in various oxidative stress-related diseases [40]. Studies had found that treatment with vitexin significantly reduced lipid peroxidation and ROS in high glucose-induced INS-1 cells, while also enhancing antioxidant levels [41]. Vitexin supplementation could also restore the decrease of Sirt1/Bcl-2 expression and inhibit the increase of caspase3 expression in vitro, exerting a protective effect against alcoholic liver injury. Its antioxidant stress mechanism may be related to Sirt1/p53-mediated mitochondrial apoptosis pathway [42]. The study also found that psoralen can play an anti-oxidative stress effect by activating the endoplasmic reticulum stress signaling pathway and promoting cell apoptosis, thereby inhibiting the occurrence of tumors [43].

### 3.4. Network Pharmacological Analysis

**Figure 3:**
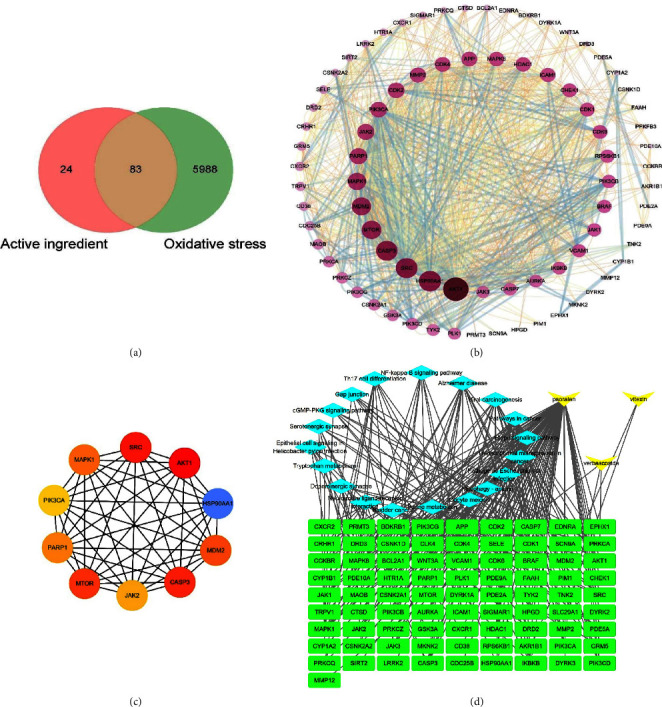
The results of network pharmacology were as follows: (a) venn diagram; (b) PPI network diagram; (c) hub target network diagram; (d) “component-target-pathway” network diagram.

After retrieving 107 component targets of verbascoside, psoralen, and vitexin, the disease targets were screened through GeneCards with a maximum degree value of 51 and a minimum degree value of 0.17. Then, 6071 targets whose correlation degree was greater than the median (1.58) were matched with component targets, and 83 potential targets were obtained after being standardized by Uniprot data (Figure 3(a)). In the PPI network (Figure 3(b)), the darker the color, the larger the volume, indicating the greater the connectivity. Using the Selectnodes option in the cytoHubba plugin window, the top 10 nodescores were obtained (Figure 3(c)). The combined analysis suggests that AKT1, HSP90AA1, SRC, CASP3, MTOR, MDM2, and PARP1 are the core targets of YWD's effectiveness and may play important roles in antioxidant stress. The “compound-target-pathway” network (Figure 3(d)) was constructed using CytoScape 3.7.2, in which the yellow inverted triangles represented active compounds, the turquoise diamonds represented signaling pathways, and green rectangles represented key target proteins. This clearly demonstrated that the active compounds in WYD could exert synergistic effects against oxidative stress by intervening in multiple targets and interacting on multiple pathways.

**Figure 4:**
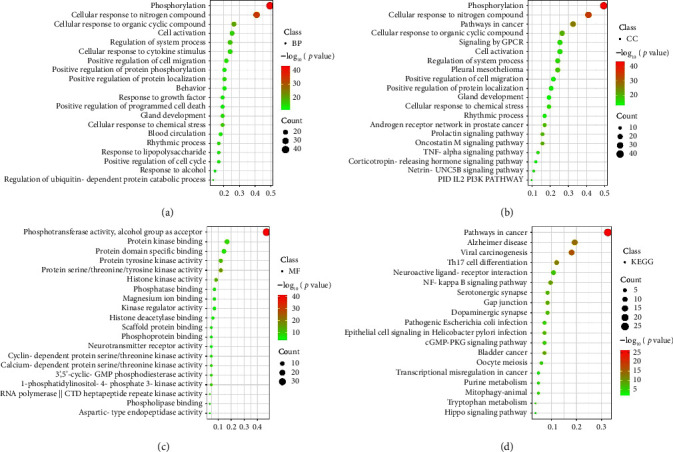
Enrichment analysis of GO ((a) BP entry; (b) CC entry; (c) MF entry) and KEGG pathways (d) of target genes of YWD for antioxidant stress.

Next, GO and KEGG functional enrichment analysis was performed on the targets related to YWD antioxidant stress through the Metascape platform, and the data were visualized (Figure 4). GO analysis showed that YWD mainly exerted its role in antistress injury, regulation of cell migration, and apoptosis through biological processes such as phosphorylation, regulation of chemical stress response, regulation of kinase activity, response to toxic substances, positive regulation of cell migration, stress response to cytokines, stress response to nitrogen compounds, Netrin-UNC5B, and prolactin-mediated signaling pathway. KEGG pathway analysis showed that YWD alleviated oxidative stress mainly by participating in Th17 cell differentiation, dopaminergic synapse, neuroactive ligand receptor interaction, and NF-ΚB inflammatory pathways.

We know that the increase of intracellular oxygen-free radicals and other active oxidizing substances will lead to oxidative stress. It had been confirmed that inflammation and cell apoptosis were the main pathological changes in the process of oxidative stress [44]. Oxidative stress inhibited the activity of Akt pathway [45], and drugs could improve the activity of caspase-3 in cardiomyocytes in an Akt-dependent manner [46]. It is evident that Akt plays an important role in the process of oxidative stress. It can also regulate NF-*κ*B signaling transduction by phosphorylating IKK*α*, and the phosphorylation of AKT and the translocation of NF-*κ*B are also key factors in apoptosis-related pathways [45]. Therefore, it is highly possible that YWD can exert its antioxidant stress function by regulating cell apoptosis through the Akt/NF-*κ*B pathway.

### 3.5. Molecular Docking Analysis

**Figure 5:**
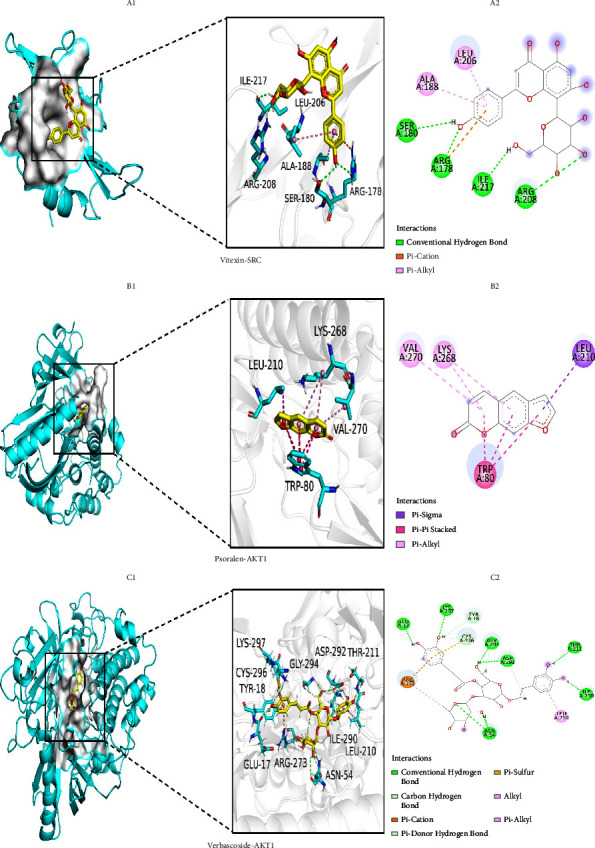
Molecular docking patterns between main core components and core targets of YWD for antioxidant stress.

To validate the findings of network pharmacology, we used molecular docking to evaluate the binding ability between the screened active drug and the core target.  Table 7 provides detailed information on the docking gene targets and compounds, showing the binding energy (kcal/mol) of the key targets and active compounds. The free binding energy of the docking results was in the range of −6.1 to −10.5 kcal/mol, indicating stable binding. Visualization of representative compound target interactions and their binding patterns using PyMoL1.7.2.1 and DiscoveryStudio2020 (Figure 5(A1, A2, B1, B2, C1, and C2)). Such as the free binding energy of vitexin to SRC is −6.2 kcal/mol, and the binding affinity is attributed to hydrogen bonding between residues of SER-180, ARG-178, ILE-217, and ARG-208, as well as hydrophobic interactions with ALA-188 and LEU-206 residues of SRC. Through hydrogen bonding, hydrophobic small molecules formed stable complexes with the active cavity of the target protein [47]. In this study, we also found that verbascoside showed strong binding activity with AKT1, CASP3, and SRC, and AKT1 was well combined with vitexin and psoralen. It can be said that molecular docking techniques provide strategies for evaluating binding patterns between herbal compounds and disease-related targets. However, it should be noted that potential compounds and targets still need to be validated experimentally.

### 3.6. Molecular Dynamics Simulation Analysis

**Figure 6:**
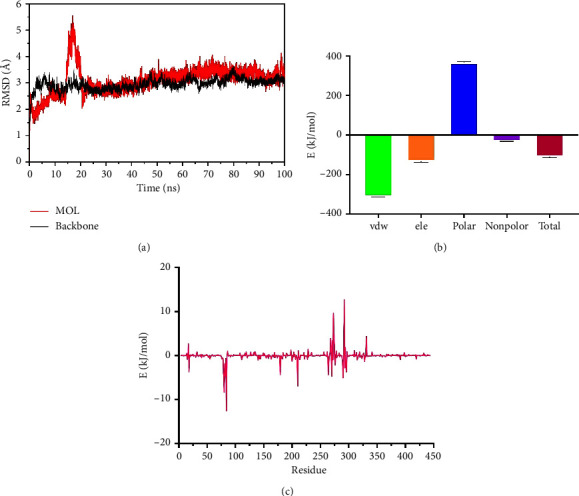
AKT1 and verbascoside of MD simulation. (a) The RMSD remained stable through the whole simulation. (b) Free energy state. (c) Free energy decomposition.

We selected AKT1 and verbascoside for molecular dynamics simulation. The Root Mean Square Deviation (RMSD) is considered as a standard for measuring the stability of the system, and the smaller the average RMSD value; the more stable the system [48]. During the 100 ns simulation, the RMSD value remained low and stable, fluctuating around 3 Å (Figure 6(a)). The results showed that the binding between the ligand and the receptor was close and the complex was stable. We took the last 20 ns of the simulation and used MMPBSA to calculate and decompose the free energy. We found that van der Waals energy is the main energy in the interaction between small molecules and proteins. Electrostatic interaction and nonpolar solvation energy are also favorable for the binding of small molecules and proteins, although polar solvation energy hinders the binding between proteins and small molecules. However, the high van der Waals energy compensates for this hindrance. The interaction energy between the protein and the ligand is −110.785 ± 3.569 (kJ/mol), which confirms the reliability of the binding (Figure 6(b) and 6(c)). We then found that ASP, ARG, and ASP correspond to the largest absolute free energy, indicating that they are key amino acids.

## 4. Conclusions

In this study, we have demonstrated that the spectral effect relationship can serve as an effective method for identifying active components in complex compound systems. After employing the ultra-high-performance liquid chromatography fingerprinting method, we calibrated 15 common peaks in 10 batches of YWD and identified verbascoside, vitexin, and psoralen among these peaks. In addition, the variation of chemical composition due to complex production conditions has resulted in different antioxidant effects of YWD batches, as indicated by the antioxidant determination results. Therefore, it is necessary to implement quality control on representative components in order to achieve better efficacy in the future. The study of spectral effect relationship based on multivariate statistical model indicated that the possibility of the identified three peaks as antioxidant stress-active compounds was discussed. Based on this, the present study employed bioinformatics methods such as network pharmacology, molecular docking, and molecular dynamics simulations to systematically analyze the antioxidant mechanisms of YWD. Our study revealed the multicomponent and multipathway action mechanisms of YWD and its potential in the treatment of antioxidant-related diseases.

## Figures and Tables

**Table 1:** Source of samples for decoction pieces of YWD.

No.	*Rehmannia glutinosa*	*Ophiopogonis Radix*	*Plygonatum odoratum*	*Glehnia littoralis*
S1	Jiaozuo, Henan 220709	Jiangyou, Sichuan 230112	Shaodong, Hunan 220707	Chifeng, Neimengo 20220916
S2	Xiangfen, Shanxi 221113	Xiaoshan, Zhejiang 220806	Dongyang, Zhejiang 220712	Chifeng, Neimengo 20220909
S3	Wenxian, Henan 230717010	Jiangyou, Sichuan 221103	Shaoyang, Hunan 230805013	Anguo, Hebei 220927
S4	Jiaozuo, Henan 220712	Mianyang, Sichuan 20221013	Shaodong, Hunan 221020	Anguo, Hebei 220602
S5	Xiangfen, Shanxi 221027	Xiaoshan, Zhejiang 221006	Shaoyang, Hunan 230703010	Laiyang, Shandong 220823
S6	Jiaozuo, Henan 220816	Mianyang, Sichuan 2022105	Dongyang, Zhejiang 220719	Laiyang, Shandong 220811
S7	Xiangfen, Shanxi 221106	Xiaoshan, Zhejiang 220718	Shaodong, Hunan 221012	Chifeng, Neimengo 20220921
S8	Wenxian, Henan 230706005	Jiangyou, Sichuan 221112	Shaoyang, Hunan 230810009	Laiyang, Shandong 220806
S9	Jiaozuo, Henan 220903	Mianyang, Sichuan 20221021	Shaodong, Hunan 221017	Anguo, Hebei 220709
S10	Wenxian, Henan 230710014	Jiangyou, Sichuan 221108	Shaoyang, Hunan 230720021	Anguo, Hebei 220611

**Table 2:** Relative retention time of common peaks in UPLC fingerprint of 10 batches of YWD.

No.	S1	S2	S3	S4	S5	S6	S7	S8	S9	S10	RSD (%)
1	0.103	0.103	0.103	0.103	0.103	0.103	0.103	0.103	0.103	0.103	0.000
2	0.163	0.164	0.164	0.164	0.164	0.164	0.164	0.164	0.164	0.165	0.000
3	0.344	0.348	0.352	0.342	0.346	0.345	0.348	0.345	0.345	0.346	0.867
4	0.516	0.517	0.517	0.516	0.516	0.516	0.517	0.517	0.517	0.518	0.193
5	0.579	0.579	0.580	0.578	0.579	0.579	0.580	0.579	0.579	0.580	0.172
6	0.710	0.710	0.710	0.708	0.708	0.709	0.710	0.709	0.709	0.709	0.141
7	0.839	0.837	0.837	0.837	0.837	0.838	0.837	0.838	0.837	0.837	0.119
8	0.865	0.865	0.865	0.865	0.865	0.865	0.865	0.865	0.865	0.866	0.000
9 (**R**)	1.000	1.000	1.000	1.000	1.000	1.000	1.000	1.000	1.000	1.000	0.000
10	1.036	1.036	1.036	1.037	1.037	1.036	1.036	1.036	1.036	1.036	0.000
11	1.084	1.084	1.084	1.085	1.085	1.084	1.084	1.084	1.085	1.084	0.000
12	1.307	1.308	1.306	1.306	1.307	1.306	1.308	1.307	1.307	1.307	0.077
13	1.442	1.439	1.438	1.442	1.442	1.440	1.442	1.441	1.442	1.443	0.069
14	1.484	1.481	1.481	1.486	1.485	1.483	1.485	1.483	1.485	1.486	0.135
15	1.575	1.573	1.573	1.579	1.578	1.576	1.577	1.575	1.578	1.579	0.127

**Table 3:** Relative peak area of common peaks in UPLC fingerprint of 10 batches of YWD.

No.	S1	S2	S3	S4	S5	S6	S7	S8	S9	S10	RSD (%)
1	0.911	0.606	1.377	2.029	2.516	1.359	1.423	3.001	1.787	1.323	44.213
2	0.363	0.224	0.658	1.012	0.820	0.642	0.851	1.628	0.971	0.656	49.425
3	0.313	0.224	0.524	0.972	0.788	0.442	0.557	1.114	0.726	0.517	45.793
4	0.388	0.158	0.489	0.503	0.991	0.624	0.844	1.744	0.933	0.710	59.214
5	0.863	0.394	1.014	1.112	1.476	1.157	1.642	3.436	2.248	1.136	58.978
6	1.669	0.571	1.487	2.552	2.148	1.304	0.702	2.220	2.083	1.167	41.824
7	0.984	0.480	0.743	1.503	1.501	0.831	0.756	1.375	1.075	0.731	35.772
8	0.307	0.190	0.400	0.555	0.717	0.303	0.450	0.766	0.486	0.374	40.220
9 (**R**)	1.000	1.000	1.000	1.000	1.000	1.000	1.000	1.000	1.000	1.000	0.000
10	0.435	0.215	0.312	0.790	0.763	0.322	0.444	0.911	0.560	0.361	46.184
11	1.497	1.301	1.214	1.819	1.129	1.009	0.939	1.048	1.081	0.892	28.412
12	0.232	0.148	0.414	0.696	0.447	0.247	0.291	0.554	0.419	0.278	44.504
13	0.236	0.136	0.142	0.219	0.281	0.205	0.524	0.363	0.182	0.159	48.980
14	0.303	0.178	0.183	0.495	0.320	0.201	0.356	0.386	0.234	0.169	38.163
15	0.019	0.010	0.033	0.061	0.027	0.049	0.065	0.140	0.097	0.052	70.909

**Table 4:** Similarity of UPLC fingerprints of 10 batches of YWD samples with consensus mode control spectrum.

No.	Similarity coefficient										
	S1	S2	S3	S4	S5	S6	S7	S8	S9	S10	Control
S1	1										
S2	0.930	1									
S3	0.967	0.901	1								
S4	0.965	0.865	0.979	1							
S5	0.919	0.805	0.969	0.967	1						
S6	0.954	0.882	0.991	0.961	0.977	1					
S7	0.860	0.821	0.927	0.874	0.925	0.957	1				
S8	0.833	0.710	0.917	0.883	0.949	0.947	0.963	1			
S9	0.914	0.791	0.964	0.937	0.965	0.979	0.950	0.98	1		
S10	0.935	0.868	0.986	0.950	0.976	0.997	0.967	0.957	0.979	1	
Control	0.962	0.894	0.993	0.968	0.975	0.998	0.957	0.942	0.977	0.995	1

**Table 5:** Results of 10 batches of YWD in vitro antioxidant activities.

No.	DPPH scavenging rate (%)	Total antioxidant value (*μ*mol/mL)
S1	69.69	1.74 ± 0.06
S2	79.01	1.26 ± 0.06
S3	53.34	1.45 ± 0.05
S4	70.27	1.18 ± 0.05
S5	65.41	0.94 ± 0.06
S6	75.48	0.95 ± 0.05
S7	82.49	1.22 ± 0.05
S8	54.91	1.10 ± 0.06
S9	82.21	1.13 ± 0.06
S10	82.39	1.75 ± 0.06

**Table 6:** GRA between of 10 batches of YWD benchmark sample and antioxidant activity.

No.	DPPH	Sort	No.	T-AOC	Sort
*X*_1_	0.812	5	*X*_1_	0.791	7
*X*_2_	0.827	4	*X*_2_	0.793	6
*X*_3_	0.710	9	*X*_3_	0.638	14
*X*_4_	0.694	11	*X*_4_	0.653	13
*X*_5_	0.760	7	*X*_5_	0.821	3
*X*_6_	0.828	3	*X*_6_	0.834	2
*X*_7_	0.850	2	*X*_7_	0.806	5
*X*_8_	0.702	10	*X*_8_	0.708	11
*X*_9_	0.862	1	*X*_9_	0.815	4
*X*_10_	0.655	14	*X*_10_	0.724	10
*X*_11_	0.655	13	*X*_11_	0.724	9
*X*_12_	0.789	6	*X*_12_	0.856	1
*X*_13_	0.670	12	*X*_13_	0.677	12
*X*_14_	0.738	8	*X*_14_	0.749	8
*X*_15_	0.614	15	*X*_15_	0.574	15

**Table 7:** Molecular docking results of main active ingredients.

Core component	Binding energy (kJ**·**mo**l**^−1^)		
	CASP3 (PDBID 2J31)	SRC (PDBID 4F5B)	AKT1 (PDBID 6HHG)
Psoralen	−6.4	−6.1	−7.8
Vitexin	−6.6	−6.2	−10.5
Verbascoside	−8.0	−7.0	−10.1

## Data Availability

All data generated or analyzed during this study are included in this paper.

## Supporting Information

Supporting Information Supporting Figure S1: The overall detection of YWD at three different wavelengths. (A) 254 nm; (B) 296 nm; (C) 330 nm.
