# Supplementary figures and images for: Screening Antioxidant Components in Yiwei Decoction Using Spectrum-Effect Relationship and Network Pharmacology
Source: J Anal Methods Chem. 2024 Oct 16;2024:5514265. doi: 10.1155/2024/5514265 (PMC11498994; doi:10.1155/2024/5514265)

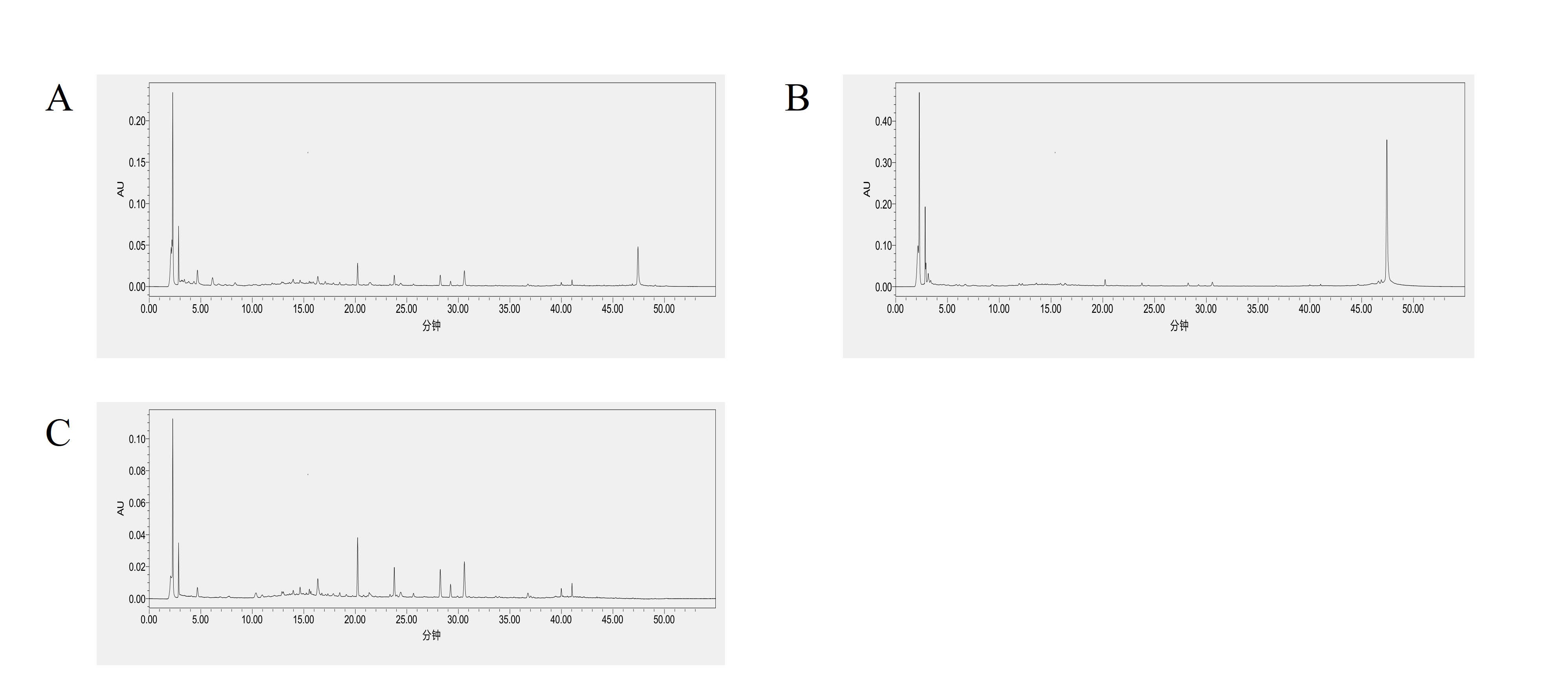

Supplement: Supporting Information — Supporting Figure S1: The overall detection of YWD at three different wavelengths. (A) 254 nm; (B) 296 nm; (C) 330 nm. [file 5514265.f1.zip › Supplementary Figure S1.jpg]
